# Development and Characterization of Powdered Antioxidant Compounds Made from Shiraz (*Vitis vinifera* L.) Grape Peels and Arrowroot (*Maranta arundinacea* L.)

**DOI:** 10.1155/2022/7664321

**Published:** 2022-04-26

**Authors:** Euzélia Lima Souza, Talita Sousa Nascimento, Camila Miranda Magalhães, Gabriele de Abreu Barreto, Ingrid Lessa Leal, Jeancarlo Pereira dos Anjos, Bruna Aparecida Souza Machado

**Affiliations:** ^1^Post-Graduate Program–Industrial Technology and Management, University Center SENAI/CIMATEC, Salvador, Bahia, Brazil; ^2^Nutrition School, Gastronomy Course, Federal University of Bahia, Salvador, Bahia, Brazil; ^3^Institute of Health Sciences, Biotechnology Course, Federal University of Bahia, Salvador, Bahia, Brazil; ^4^SENAI Institute of Innovation in Advanced Health Systems (ISI SAS), University Center SENAI/CIMATEC, National Service of Industrial Learning–SENAI, Salvador, Bahia, Brazil; ^5^Laboratory of Applied Research in Food and Biotechnology, University Center SENAI/CIMATEC, National Service of Industrial Learning–SENAI, Salvador, Bahia, Brazil

## Abstract

Grapevine (*Vitis vinifera* L.) is a plant containing many phenolic compounds, mostly distributed in the peel, pulp, and seeds. This study evaluates the centesimal composition and bioactive compounds in Shiraz grape (*Vitis vinifera*) peels using spectrophotometric and UHPLC techniques and develops different formulations of compound powders from the peels and arrowroot using conventional drying technology. The results demonstrate that Shiraz grape skin contains significant amounts of insoluble fiber (15.3%), phenolics (157.09 ± 6.96–149.11 ± 9.27 mg GAE g^−1^), and flavonoids (0.75 ± 0.50–2.00 ± 0.50 mg QE g^−1^), with excellent antioxidant capacity observed in the alcoholic extracts. The phenolic content in the developed powdered compounds ranged from 128.32 to 139.70 mg GAE g^−1^. In general, the compounds showed good antioxidant capacity (IC50 = 0.17 to 0.19 *μ*g mL^−1^). According to the chromatographic evaluation, it was possible to quantify gallic acid, catechin, and epicatechin, the latter of which was found in the largest quantities in the six formulations. The EV5 formulation was the most efficient in terms of phenolic compounds and protein amounts. This formulation's composition and low cost could make it viable for use in the food industry.

## 1. Introduction

Grape is one of the most valued fruits on the planet [1] and can be consumed fresh or used for the production of wine, juices, and jellies. The nutritional benefits of grape for human health have been widely studied due to the antioxidant phenolic compounds present in the fruit [2, 3]. The dynamism of the Brazilian wine industry during recent years has led the country to be considered one of the greatest world producers of fine wine. In Brazil, the regions with the highest production of good quality wines are located in the South and Northeast, specifically in the São Francisco Submediant Valley Region, which is ranked second nationally as a producer of fine wines made with the species *Vitis vinifera* L. [4]. *Shiraz* is the main cultivar for red wines in this region, and its residues are considered to be potential sources of significant phenolic compounds, especially stilbenes and flavonoids [5, 6], as well as promising technological ingredients containing more than 60% dietary fiber [7], including insoluble dietary fiber (such as cellulose and hemicellulose) and soluble dietary fiber (such as *β*-glucans, pectins, and gums) [8].

The industrial process for grape generates significant quantities of bagasse, corresponding to between 20% and 30% of the total volume [9, 10]. These residues are composed of bark, seeds, and stems that still contain, even after processing, high levels of phenolic compounds, flavonoids, tannins, and saponins and are generally transformed into fertilizers and animal feed or are discarded in nature. However, despite being biodegradable, these compounds require a minimum time for degradation, and their use in new approaches could represent a great opportunity for the development of new products. In this way, reuse of these residues would help reduce waste, thereby adding economic, social, environmental, and nutritional value to the production of products for different industries, especially food [9, 11].

Grape skins are a source of bioactive compounds, including phenolic compounds [12, 13] that have antimicrobial [5, 14], anti-inflammatory [15], and antioxidant properties [16, 17]; anthocyanidins and anthocyanins, which are natural dyes that have antioxidant properties, including lipoperoxidation inhibition and antimutagenic activity [18, 19]; and flavonoids, which have been widely studied for their beneficial effects on human health and are considered potential ingredients in food formulations and supplements [20–22].

Due to their high moisture content (around 80%), fresh fruits and vegetables are highly perishable and deteriorate rapidly [23, 24]. In this way, reducing the moisture content and, consequently, the water activity of fruits and vegetables would reduce microbial activity and minimize the physical and chemical changes that occur during food storage [25]. In the scientific community, some research has focused on the use of residues from the agrifood industry, such as rice bran, by-products of processed fruit pulp, bean flour, and grape marc flour, which is used in food products such as baking, confectioneries, and dairy [26–30].

Despite the aforementioned benefits, it should be noted that the incorporation of raw materials with high fiber content and a high percentage of moisture alongside a low pH in the formulation of food products could interfere with the technological parameters (moisture, pH, and texture); thus, adaptations are needed in the manufacturing processes or in the formulation of the products. In this way, reducing the moisture content and, consequently, the water activity can reduce microbial activity and minimize the physical and chemical changes that occur during storage [26]. One option to minimize such interference is the application of existing artificial drying techniques, among which conventional drying, also known as hot-air drying, is the most economical technique and has been widely adopted in the food industry [31]. Hot-air drying is a low-cost process that generates more stable products during storage [31, 32].

Another way to reduce these problems in the technological standards of the products developed is the addition of components rich in starch, such as arrowroot (*Maranta arundinacea* L.), which is an herbaceous plant belonging to *Marantaceae* whose rhizomes contain starch reserves. This plant is suitable for all combinations with water and milk and, therefore, works in countless gastronomic preparations [33, 34]. Arrowroot could minimize the interference that occurs in products rich in fiber with a low pH, as arrowroot impacts the texture of food, thickening and increasing the concentration of solids [35]. Consequently, arrowroot may offer viable technological properties as a solution to bakery and food application.

Under the background of an integrative and innovative approach, the search for a better quality of life and conscious consumption, and increased understandings of rights, social and environmental responsibility in the acquisition of food has become a topic of great magnitude and has been discussed worldwide with great attention in recent years [36–39]. Current analyses highlight global consumption dynamics based on the current impositions and directions of food consumers. New perspectives on food are based on the consumption of more natural foods, foods rich in nutrients, and foods that offer health benefits while retaining good sensory aspects [38]. The food and beverage sector is converging on the creation of products that can meet the trends and demands of consumers. The main trends include sensorial experiences and pleasure, health and well-being, convenience and practicality, reliability and quality, and sustainability and ethics.

In this context, the present study aims to develop and characterize different formulations of powdered antioxidant compounds extracted from Shiraz red grape skins (residue from the wine and grape juice industry) and arrowroot (*Maranta arundinacea* L.) using conventional drying technology.

## 2. Materials and Methods

Shiraz grape pomace from the August 2018 harvest in the “São Francisco” Sub-Middle Valley region was used. Manual separation of the peels, seeds, and stalks was performed. Then, the shells were vacuum-packed and stored below freezing at a temperature of −21°C until use.

Deionized water obtained using a Milli-Q system (Millipore, Molsheim, France) was used. Folin-Ciocalteu reagents, gallic acid, and 2,2-diphenyl-1-picrylhydrazyl (DPPH) were purchased from Sigma-Aldrich. The following reagents of HPLC grade were used: Methanol, DMSO (Sigma-Aldrich Chemical Co., St. Louis, MO, USA), acetic acid (Dinamic), and grain alcohol (92.8%, Anidrol). The syringe filter was a PES Millipore (Filtrilo, Paraná, Brazil). The standards of caffeic acid (CAS number 331-39-5), gallic acid (CAS number 149-91-7), *trans*-cinnamic acid (CAS number 140-10-3), crystalline p-coumaric acid (CAS number 501-98-4), hydrated catechin (CAS number 225937-10-0), biochanin A (CAS number 491-80-5), ellagic acid (CAS number 476-66-4), epicatechin (CAS number 490-46-0), formononetin (CAS number 485-72-3), isoliquiritigenin (CAS number 961-29-5), myricetin (CAS number 529-44-2), naringenin (CAS number 67604-48-2), quercetin (CAS number 117-39-5), resveratrol (CAS number 501-36-0), rutin trihydrate (CAS number 250249-75-3), and piceatannol (CAS number 10083-24-6) were acquired from Sigma-Aldrich Chemical Co. (St. Louis, MO, USA); kaempferol (CAS number 520-18-3), kaempferide (CAS number 491-54-3), and *trans*-ferulic acid (CAS number 1135-24-6) were obtained from Fluka.

### 2.1. Development of Powdered Antioxidant Compounds

The fresh grape skins were previously dried in an air circulation oven at 50°C for 2 hours until the samples reached humidity below 20%.

The samples of the compounds were named EV1, EV2, EV3, EV4, EV5, and EV6. Different amounts of powdered grape skins (70 and 60%) and arrowroot (30 and 40%) were used by drying the mixtures in an air circulation oven (Quimis, Q314M222, Brazil) for 1 hour. Different drying temperatures (40, 50, and 60°C) [40] were also used, as shown in Table 1.

Subsequently, the formulations were ground in an ultracentrifugal knife mill (Retsch, Haan, Germany) and sieved (<0.5 mm) using a sieve shaker (AS 200, Haan, Germany). The materials were stored under vacuum in individual metallic pouches.

### 2.2. Characterization of the Centesimal Composition of Shiraz Grape Skins and Powder Compounds

Moisture and ash contents were determined using the gravimetric method [41, 42]. The crude protein was quantified by the micro-Kjeldahl method through the determination of total nitrogen [42, 43]. Total lipids were extracted and quantified according to Bligh and Dyer's methodology [44]. The fibers were determined in an ANKOM automatic fiber analyzer [45] only for the grape peel sample due to the granulometry of the compost powder developed. The water activity (aw) of the samples was analyzed using a decagon (LabMaster-aw, Novasina, Brazil) at a temperature of 25°C. The content of total soluble solids (STT) was determined by reading a portable refractometer with the samples diluted in distilled water (1 : 10 w/v). The pH was determined using a digital bench pH meter (FiveEasy Plus, Mettler Toledo, Switzerland). Total titratable acidity was determined via titration with NaOH (0.1 N) and expressed as the percentage of citric acid [42]. Instrumental color analysis was performed using an *L∗*, *a∗*, *b∗*, *C*, and H° system in a colorimeter (CR-410, Konica Minolta, Japan).

The hygroscopicity of power for the sample compounds was determined according to methodology described by Moraes [46]. This value was calculated according to the following equation:

(1) $$\begin{array}{c} \%\text{ hygroscopicity }=\;\frac{\left(F_{w}-I_{w}\right)}{I_{w}}\times 100, \end{array}$$

where *I*_*w*_ is the initial plate weight + powder (g) and *F*_*w*_ is the plate weight + powder in balance (g).

### 2.3. Evaluation of the Bioactive Properties of Grape Peels and the Formulations Obtained

#### 2.3.1. Obtaining the Extract

Three extracts of Shiraz grape skins with solvent variations were obtained: an aqueous extract (EA), an extract of 50% cereal alcohol (EAC50), and an extract of 80% cereal alcohol (EAC80). The skins were crushed with the solvent in a 1 : 5 m/v ratio. Then, the mixture was submitted to an ultrasound bath (S40H, Elma Sonic, Germany) for 30 min/60°C. Next, the mixture was homogenized in a shaker type incubator (MA420, Marconi, Brazil) for 120 min (180 rpm), filtered, and concentrated in a sample concentrator (MiVac Concentrator, Genevac, Canada) at 50°C. The extract was then stored at a freezing temperature (−27°C).

For the 6 extracts of the powdered compounds, variations of the solvents were used. 50% cereal alcohol was the most efficient in extracting the compounds. The samples were mixed with the solvents in a proportion of 1 : 5 m/v. The same procedure was followed to obtain the grape peel extracts [47].

#### 2.3.2. Determination of the Content of Total Phenolic Compounds

To quantify the phenolic compounds from the extracts of the grape peels (EA, EAC50, and EAC80) and the formulations obtained (EV1, EV2, EV3, EV4, EV5, and EV6), the peels were diluted in 50% ethanol at a concentration of 1 : 2 m/v. The analysis was performed according to the spectrophotometric method with a Folin-Ciocalteu reagent, as described by Singleton et al. [48], using gallic acid as a standard. The absorbance reading was done at 765 nm at room temperature, and the results of the total phenolic compounds were expressed as the gallic acid equivalent (milligrams of GAE per gram of sample).

#### 2.3.3. Determination of the Content of Total Flavonoids

The flavonoid content determination of samples was performed using a spectrophotometer (Lambda 25 UV/Vis Systems, PerkinElmer, Washington, USA) at 415 nm. The solution was prepared using aluminum chloride at 2.0% in methanol [49] in a 1 : 1 solution. The same procedure was performed using known solutions of quercetin standard to elaborate a standard curve (*y* = 0.0311*x* + 0.0259; *R*^2^ = 0.9987). Furthermore, a blank sample was prepared under the same conditions and the quantity of flavonoid content was expressed as quercetin equivalents (EQ) (mg EQ g^−1^). All the analyses were executed in triplicate.

#### 2.3.4. Total Anthocyanins

Determination of the anthocyanin content was carried out according to the methodology described by Francis [50]. About 0.5 g of the sample (grape peels and 6 different formulations) was homogenized with 30 mL of the extraction solution (95% ethanol + 1.5 N HCl and 95% methanol at a ratio of 85 : 15 v/v) and maintained at rest for 12 h at 4°C. Then, the material was filtered and kept at rest for 2 hours at room temperature, and readings were performed on a spectrophotometer (LAMBDA 25 UV/Vis Systems, PerkinElmer, USA) at 535 nm.

#### 2.3.5. Determination of the Antioxidant Capacity

The antioxidant activity was evaluated via the DPPH method (2,2-diphenyl-1-picrylhydrazyl) using a spectrophotometer (LAMBDA 25 UV/Vis Systems, PerkinElmer, USA) at 517 nm, according to the methodology described by Molyneux [51] and Brand-Williams et al. [52] with adaptations. Six dilutions were prepared from the extracts obtained. Then, a 1.0 mL aliquot of each dilution was transferred to tubes containing 3.0 mL of the DPPH ethanolic radical solution (0.004%). After 30 minutes of incubation in the dark, readings were performed. The result for antioxidant activity was given based on the corresponding amount of the sample (mg g^−1^) necessary to reduce the initial DPPH radical concentration by 50% (IC_50_).

#### 2.3.6. Identification and Quantification of Bioactive Analytes in Powdered Compounds by UHPLC

Analyses of the bioactive compounds were performed using an Ultra-High Performance Liquid Chromatograph (UHPLC) (Thermo Scientific™, Dionex™ UltiMate™ 3000 series) equipped with a pump (Thermo Scientific™, Dionex™ UltiMate™), automatic injector (Thermo Scientific™, Dionex™ UltiMate™, WPS 3000 TSL Analytical), chromatographic column oven (TCC 3000SD), and diode array detector (DAD) (Thermo Scientific™, Dionex™ UltiMate™, VWS 3400RS). Analyte separation was performed using a NUCLEODUR® 100-5 C18 ec column (150 × 4 mm ID; particle size 5 *μ*m) and a ZORBAX Eclipse Plus C18 precolumn (4.6 × 12.5 mm) (Agilent, USA).

The analysis conditions included an elution gradient of the mobile phase composed of 5% acetic acid (solvent A) and methanol (solvent B) in different proportions, with a total analysis time of 42 min (0 to 35 min (0–92% B), 35 to 40 min (92–0% B), and 40 to 42 min (0% B)). The injection volume of the samples and standard solutions was 20 *μ*L, and the flow rate of the mobile phase was kept constant (1 mL min^−1^). The oven for the column was operated at a temperature of 40°C. The bioactive compounds were detected using the respective wavelengths for each analyte: 280 nm for gallic acid, *trans*-cinnamic acid, biochanin A, catechin, epicatechin, formonometin, isoliquiritigenin, myricetin, and naringenin; 300 nm for p-coumaric acid; 320 nm for caffeic acid, *trans*-ferulic acid, ellagic acid, piceatannol, and resveratrol; and 370 nm for rutin trihydrate, isoliquiritigenin, myricetin, naringenin, quercetin, kaempferide, and kaempferol.

To analyze the bioactive compounds, a stock solution was prepared containing a mixture of all analytes (caffeic acid, gallic acid, p-coumaric acid, *trans*-cinnamic acid, *trans*-ferulic acid, biochanin A, catechin, epicatechin, formonometin, isoliquiritigenin, myricetin, naringenin, quercetin, resveratrol, ellagic acid, piceatannol, and rutin trihydrate), each at a concentration of 500 mg L^−1^ and solubilized in high purity methanol or DMSO.

Bioactive compounds in the samples were identified by comparing the retention times and UV-VIS spectra between the standards and samples. To quantify the compounds, analytical curves were constructed at concentrations varying between 0.5 and 25 mg L^−1^ (*n* = 5) from successive dilutions of the stock solution in methanol. For the analytical curves, coefficients of determination (*R*^2^) greater than or equal to 0.9999 were obtained, showing good linearity between the concentration of the analytes and the areas of the chromatographic peaks.

Samples of the powdered compounds were solubilized in methanol (50%) (varying between 4.0 and 5.2 mg mL^−1^). Subsequently, all samples and standard solutions were filtered on a 13 mm, 0.22 *μ*m PES Millipore filter (Filtrilo) before injection into the chromatographic system. All analyses were performed in triplicate.

#### 2.3.7. Statistical Analysis

The results of this study were expressed as the mean ± standard error of mean (SEM). The statistical analysis of the results was performed using the Statistica 6.0 software from StatSoft (Tulsa, USA). A one-way ANOVA and the Tukey test (95% confidence level) were used to identify the differences between the centesimal composition and concentrations of the phenolic compounds and flavonoids, while the concentrations of compounds were determined by UHPLC. The antioxidant activity in the extracts was determined for the Shiraz grape peels and the powder compound. In all statistical procedures, the level of significance was set at *p* < 0.05.

## 3. Results and Discussion

### 3.1. Characterization of the Centesimal Composition of Shiraz Grape Skins and Powder Compounds

Table 2 shows the results for the centesimal composition of the grape peels. The moisture (68.82 ± 1.07%) observed on the Shiraz grape skins was higher than that observed by Ribeiro et al. [41] (65% and 51%) from two wineries in the São Francisco Valley. Regarding the ash parameter, the result found in this study for fixed mineral residue corresponding to the inorganic elements present in food (4.12%) was higher than that found by Hernandes [43] and Lopes [53] for Cabernet Sauvignon grape pomace (2.04%), conventional grape waste (0.75%), and organic grape waste (0.63%). A pH of 3.54 ± 0.05 was determined for the Shiraz grape skins, which was lower than the pH levels found by Oliveira et al. [54] in a study exploring the impacts of climate on the physicochemical composition of Shiraz grapes at low and high altitudes in tropical cultivated regions of Brazil. This value is also lower than the values found in the study by Souza et al. [47], who examined varieties such as Italy (4.53), Brazil (4.13), Ruby (4.66), Thompson (4.09), and Niagara (4.03) grapes, and very close to the values found by Oliveira [6] in a study on Shiraz grapes grown in the same region (3.68 and 3.98). According to Sigler [55], grapes grown in tropical climates (with average temperatures close to 30°C) are generally more acidic than those planted in cold climate regions. Factors such as pH and volatile acidity, as well as other chemical compounds such as anthocyanins, phenolic compounds, and tannins, are responsible for the sensory and color attributes of red grape wines and juices [56]. The acidity content of a food is related to the presence of organic acids in the sample. Tartaric, malic, and citric acids are the main components responsible for the acidity of grape must, and phenolic acids contribute to this fraction. Acidity is an important parameter in food, not only because of the flavor characteristics it adds to the product but also because acidity is an intrinsic factor in the control of microorganisms [57]. The Shiraz grape peel in this study had total titratable acidity (20.7%) lower than the values found for bagasse [58] of the cultivars Chardonnay (37.60%) and Merlot (34.02%). However, this value for Shiraz was lower than the total acidity of the cultivar Isabel (15.58%) provided in a study by Acunha et al. [40].

Shiraz grape skin was found to have higher lipid content (1.48%) than the varieties Italy, Brazil, Rubi, Thompson, and Niagara (0.34%, 0.33%, 0.35%, 0.36%, and 0.36%), respectively, according to results of Souza et al. [47]. However, the lipid content of Shiraz was lower than that presented by Deng, Penner, and Zhao [59] in grape skins from Cabernet Sauvignon (6.33%), Merlot (3.35%), and Pinot Noir (4.74%). The lower amount of lipid content in the studied matrix is justified by the fact that lipid quantification was performed only on the peel, although the highest content of lipids in vinification waste is found in the seeds (between 10% and 16%) [60].

The protein corresponded to 2.32 ± 0.33%, a value higher than that found by Ribeiro et al. [41] (0.40%) in Shiraz grapes from the same locality and lower than the values in some other studies, which ranged from 9.4% to 10.72% [61, 62]. Proteins constitute an important portion of the nutritional composition of grape residue. In general, there is a predominance of the amino acids glutamine and glutamic acid, followed by intermediate portions of leucine and lysine, while cystine and methionine present the lowest contents [62].

The total soluble solids content (1.9 °Brix) found in this study was lower than those found by Brazil et al. [58] (18 °Brix). Souza [63] observed a decrease in the liquid mass of grapes during vinification, which may result in the concentration or loss of some compounds to the by-products generated and may, therefore, justify the low value found in this study.

The fibers of the studied grapes contained 4.5% hemicellulose, 13.4% cellulose, and 15.3% lignin, as shown in Table 2. Significant insoluble lignin fiber content was detected, especially since the whole pomace was not analyzed, only the peels. The chemical composition of grape fiber is known to vary according to the cultivar, climate, and processing conditions [62]. Thus, the results demonstrate that the Shiraz variety could be a promising source of fiber, as confirmed by Amorim [64].

Table 3 shows the colorimetric characterization results for grape peels. It can be seen that, in this study, the Shiraz grape skin presented low luminosity (*L∗* = 30.18 ± 0.70), a characteristic expected for red grape skins. The Shiraz grape skins also presented a more intense tone compared to the parameters obtained by Lopes [53], which quantified the luminosity in conventional *Vitis labrusca versus Concord* (42.69) and organic (60.94) varieties.

### 3.2. Evaluation of the Bioactive Properties of Grape Peels and the Formulations Obtained

Table 4 shows the results of bioactivity compounds of grape peels, submitted to different solvents. The contents differed according to the solvent used. The extracts in 80% and 50% ethanol achieved greater extraction capacity with higher contents of total phenolics (157.09 ± 6.96 and 149.11 ± 9.27 mg GAE g^−1^, respectively), whereas the water-based solvent was not as efficient in extraction (55.84 ± 3.74 mg GAE g^−1^). Several studies in the literature analyzed phenolic content in grape extracts and their derivatives [6, 65–67]. However, the wide variety of species, different methods used for extraction, and various types of solvents in these studies make it difficult to compare their results.

The 50% ethanolic extract achieved the greatest extraction capacity (2.00 ± 0.09 mg QE g^−1^) (García-Lomillo et al.) [68] for pomace from several red grape cultivars of Pinot Noir, Isabel, Sangiovese, Negro Amaro, Cabernet Sauvignon, and Primitivo (56 ± 13, 156 ± 25, 206 ± 13, 131 ± 33, 252 ± 26, and 165 ± 19, respectively, with 161 mg QE/g^−1^ as the average) from the São Francisco Valley Region for flavonoids, albeit with a lower value than the values found in the study of Rockenbach et al. [69].

The extract in a solution of 50% ethanol showed the best antioxidant capacity (IC_50_ 0.17 ± 0.01 *μ*g mL^−1^). This capacity was better than that reported by Silva et al. [70] in Portugal (IC_50_ 0.73 ± 0.04 *μ*g mL^−1^ for the grape skin extract *Touriga*) using 50% ethanol as the extracting solvent. The aqueous extract, which was polar, presented the lowest antiradical activity (IC_50_ 0.73 ± 0.41 *μ*g mL^−1^). In general, this result was expected for antioxidant activity and is a function of the polarity of the solvents. The lower the IC_50_ value is, the higher the product's antioxidant activity will be and the lower the quantity of extract will be needed to inhibit the activity of free radicals by 50%.

The result for the total anthocyanins in Shiraz grape skins was 1.53 ± 0.41 mg g^−1^, similar to the values found in the study of Oliveira [6] in waste from the São Francisco Valley (0.715 mg g^−1^) and lower than the values observed by Hernandes [43] in Cabernet Sauvignon grape pomace (472.53 ± 0.22 mg g^−1^).

**Figure 1:**
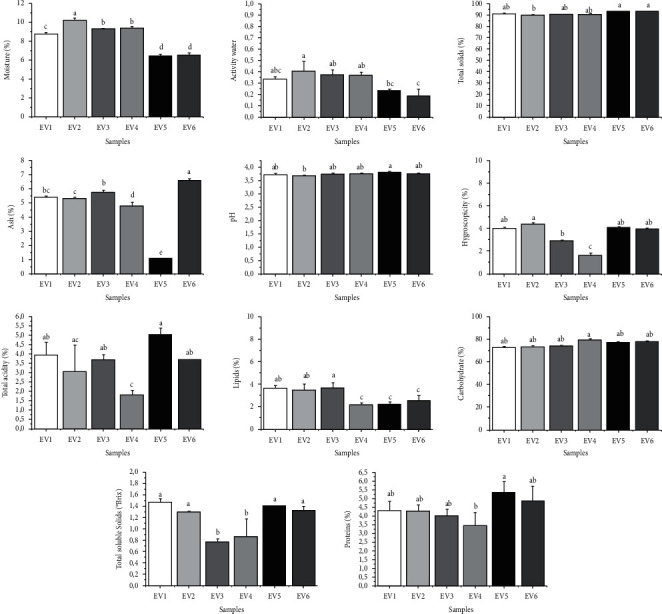
Centesimal composition of powdered compounds from Shiraz grape peels and arrowroot. Statistical analysis: values showing the same letter in the same analysis do not indicate significant differences (*p* > 0.05) based on a Tukey test at a 95% confidence level.

When considering 1 kg of the frozen bagasse husks and using conventional drying methods, the relative yield of the raw material was 255 g for dehydration at 60°C, 260 g at 50°C, and 268 g at 40°C. After adding 30 to 40% arrowroot, the final yields became 330.5 and 357 g, respectively. It was not possible to compare these results with the values in the study of Oliveira [6], because that study used the whole pomace (including peel and seeds), obtaining approximately 321 g of processed flour for 1 kg of fresh pomace, with yields close to 32%. However, our study worked only with grape peel and arrowroot.  Figure 1 presents the centesimal composition of powdered compounds from the Shiraz grape peels and arrowroot.

For the physicochemical characterization of the powdered compounds, the drying temperature was found to influence some parameters. For compounds EV5 and EV6, which received heat treatment at 60°C, *A*_w_ (0.235 ± 0.113 and 0.188 ± 0.06) and moisture (6.43 ± 0.1 and 6.52 ± 0.20) were lower relative to the compounds exposed to lower temperatures. EV3 and EV4 (50°C) had *A*_w_ of 0.374 ± 0.04 and 0.369 ± 0.300 and moisture of 9.28 ± 0.06 and 9.38 ± 0.16, while EV1 and EV2 (40°C) had *A*_w_ of 0.336 ± 0.018 and 0.410 ± 0.08, respectively, and moisture of 8.73 ± 0.20 and 10.22 ± 0.21. It was observed that higher temperatures produce a higher drying rate and, consequently, yield a reduction in the moisture ratio [40]. This phenomenon occurs due to an increase in the rate of heat supplied to the compounds and the acceleration of water migrations from their interior. There is an inverse relationship between increases in the process temperature and decreases in moisture content. There are no established parameters for identity or quality standards for grape flour [71]. However, for flour in general and flour of other origins, such as maize and carob flour, the maximum permitted humidity limit is 15%. Thus, the powdered compounds produced in this study were within the standards established by Brazilian legislation for flour [71], ranging between 6.43% and 10.22%. Oliveira et al. [54] obtained moisture values of 7.50% for grape seed meal and grape skin, and Bampi et al. [72] developed Japan grape flour with 19.08% moisture. The variation in these results could be influenced by several factors, including cultivar, process conditions, and especially the technological processes used to obtain these compounds.

The pH generally did not oscillate among the compounds analyzed, instead remaining between 3.68 ± 0.04 and 3.81 ± 0.03, which are close to the values found in the study of Bender et al. [29] (3.51 ± 0.02) for formulations with acidic characteristics. Bagasse has a low pH due to the high content of organic acids present [73], mainly tartaric, malic, and citric acids. According to Selani et al. [74], low humidity and low pH decrease the risk of enzymatic and nonenzymatic reactions and microbiological contamination, which can hinder the development of microorganisms because fungi generally prefer an acidic pH (4.5–5.0), and bacteria prefer an almost-neutral pH (6.5–7.0) [75]. Variation in protein content was observed between 3.43 ± 0.75 and 5.35 ± 0.62%, which are values lower than those found in the study of Kruger et al. [76] for residues from Serra Gaucha of the cultivars Merlot, Tannat, and Isabel (13.3 ± 0.11, 6.0 ± 0.6, and 18.5 ± 0.8%, respectively) and very close to the content found by Ribeiro [77] (5.32%) for Cabernet Sauvignon grapes. According the Resolution of the Collegiate Board of Directors [78], for flour to be considered a source of protein, it needs to have a minimum content of 6 g of protein per 100 g. The compound that came closest to the parameter considered essential was the compound EV5 (5.35 ± 0.62%).

The ash content observed in the formulations ranged from 4.81 ± 0.23 to 6.77 ± 0.14%, which are values greater than those found by Oliveira et al. [79] (2.04 and 3.69%), close to those observed by Kruger et al. [76] (6.0 ± 0.1, 6.9 ± 0.3, and 3.9 ± 0.3% for Merlot, Tannat, and Isabel cultivars, respectively), and lower than those observed for Shiraz grapes (7.0%) by Strapasson [80].

The low content of lipids found in the powdered compounds in this study (ranging from 2.15 to 3.99%) was associated exclusively with the lipid fraction of the grape pomace skin, which, in turn, was reduced in the lipids compared to the whole pomace containing seeds and stalk. The lipid content of the pomace can range between 10% and 16%, depending on the species [81]. García-Lomillo et al. [68] found 3.69% lipids in a product from grape marc skins, corroborating the values found in this study.

The total acidity of the extracted compounds presented variation between 1.82 ± 0.23 and 5.04 ± 0.32%, a value compatible with that found for grape flour by Hernades [43] (2.56%). The total soluble solids (°Brix) of the compounds oscillated between 0.77 ± 0.06 and 1.47 ± 0.06, values that are low when compared to natural grapes and their derivatives. In a study by Favero et al. [82], Shiraz grapes presented total soluble solid values of 18.84 and 21.48 °Brix for the 2005 and 2006 harvests, respectively. This study used samples of fermented Shiraz grape skins from the winemaking industry. After the winemaking process, sugars are reduced due to their consumption by microorganisms during alcoholic fermentation, thereby justifying the low content of these solids in the samples.

The carbohydrate values of the compounds were calculated based on difference, with values observed between 75.50 ± 1.62 and 80.28 ± 1.35%, higher than those found by Strapasson [80] for the 2012 and 2013 harvests of the Tannat cultivar (59 g/100 g) and for Shiraz and Bordeaux (55 g/100 g). Notably, the values found are associated with the total carbohydrate content of grape and arrowroot skins. Grape marc, as a by-product of wine and juice manufacturing, may present differences in composition depending on the industrialization methods employed in the pressing and must fermentation stages. The main differences between the results obtained in this study can be explained by the composition of the materials (grape and arrowroot skins) in comparison to the whole pomace (skin, seeds, and stalk), as well as the differences between the varieties of grapes, their states of ripeness, and so forth.

The color parameters of the obtained compounds are shown in Table 5. The compounds presented inherent coloration in products supplemented with red grape peel, which varied the values of *L∗* between 45.82 ± 0.29 and 51.43 ± 0.45. These are values close to the center of the scale and indicate medium brightness. On the *b∗* spectrum (blue), there was a slight predominance of violet, with values observed between 2.08 ± 0.02 and 2.44 ± 0.10, while in the *a∗* spectrum, the values (7.75 ± 0.06 to 8.44 ± 0.08) intensified red over yellow coloration. However, according to angle H°, which oscillated from 15.07 ± 0.24 to 17.09 ± 0.36, the coloration remained close to a “Bordeaux” tone. Thus, the compounds presented Bordeaux coloration with medium intensity. The extracted compounds presented darker tones than the values found by Bender et al. [29] (*L∗* 39.67 ± 6.28) in a study evaluating grape skin flour from grape pomace of the cultivar Marselan. In a study on organic grape skin flour (*Vitis labrusca*), Abreu et al. [83] observed values of *L* = 25.76 ± 0.09, *a∗* = 8.86 ± 0.16, and *b∗* = 0.79 ± 0.01, with the *a∗* value very close to the values found for the compounds in this study. Notably, the visual differences between the various formulations of the six compounds studied are subtle because the chromaticity variation interval was small. The dark coloring of an ingredient, in some cases, can limit that ingredient's use in food products [84].

Based on the aforementioned evaluation results for the bioactive compounds of the grape peel extracts, 50% cereal alcohol was selected as the best extracting solvent, as this solvent was able to recover more chemical compounds from the flavonoid group, thereby helping to obtain more compounds that offer antioxidant action. Thus, to obtain the extract from the formulated powdered compounds, 50% cereal alcohol was used as the solvent (Table 6).

The phenolic content in the compounds ranged from 128.32 to 139.70 mg GAE g^−1^. These are significant results when compared to those found by Kruger et al. [76] in grape flour of *Merlot* and *Tannat* (1.723 ± 263 and 1.884 ± 221 mg GAE g^−1^) and inferior to those of the Isabel cultivar (912 ± 77 mg GAE g^−1^). The compounds EV1, EV3, and EV5 obtained slightly higher concentrations of phenolics, possibly because they included a higher percentage of grape peel. As the concentration of peels in the samples increased, it was possible to observe an increase in antioxidant capacity. The phenolic compounds present in these residues have high free-radical scavenging capacity and, therefore, are important sources of primary antioxidant components, which are of great importance for the food industry [71].

The content of total flavonoids in the samples varied from 3.31 to 4.17 QE g^−1^. The compound EV5 presented the highest content. In natural extracts, flavonoid content is closely related to antioxidant potential. In this study, the compounds that obtained the best DPPH radical scavenging capacity were EV1, EV2, and EV4 (IC_50_ 0.17 ± 0.02, 0.17 ± 0.02, and 0.16 ± 0.02 *μ*g mL^−1^, respectively). The results of this study were better than those presented by Ribeiro [77], which reported IC_50_ values between 2.58 ± 0.07 and 2.70 ± 0.05 *μ*g mL^−1^ for the bagasse varieties *Cabernet Sauvignon* and *Merlot* and a Mix (composed of *Bordeaux* (65%), *Isabel* (25%), and *BRS Violeta* (10%)). The antioxidant compounds present in grapes are preserved in the skins after the winemaking process. This was confirmed by the results for the six compounds of Shiraz grapes analyzed in this study, which were observed to have satisfactory antioxidant characteristics. The results obtained using the DPPH method may be related to the presence of phenolic compounds such as phenolic acids and flavonoids. The antioxidant capacity under the DPPH method has been presented in several ways, and the lack of standardization among the results makes it difficult to compare the antioxidant capacity between different samples [59].

The compounds presented anthocyanin contents that varied between 1.16 ± 0.15 and 1.49 ± 0.03 mg g^−1^. These quantities are lower than those observed by Bennemann et al. [85] (15.78 and 114.67 mg 100 g^−1^) for flour from the grape pomace of *Cabernet Sauvignon* and *Sangiovese*. These lower results could be attributable to the thermal treatment the grapes were submitted to. However, this process cannot break the covalent bonds of the phenolic compounds, leading to losses in the quantities of these compounds (primarily due to the degradation of anthocyanins) [86]. Anthocyanins belong to the flavonoid group and are the primary compounds in dark grape skins.

### 3.3. Identification and Quantification of Bioactive Analytes in Powdered Compounds by UHPLC

Three substances were identified using UHPLC, and their concentrations are summarized in Table 7. Gallic acid, catechin, and epicatechin were found in all six formulations. Epicatechin was found in the highest amounts in all six formulations. Li et al. [87] analyzed eleven grape cultivars and noted differences in the variation of the phenolic profiles, finding lower values than those observed in our study for epicatechin (0.07 mg g^−1^) in Sangiovese grapes. A study by Bennemann et al. [85] characterized bioactive compounds and antiradical activity in grape marc flour from cultivars of Cabernet Sauvignon, Merlot, Sauvignon Blanc, and Sangiovese (*Vitis vinifera*), dehydrated under 45°C air circulation (oven or freeze-dried), finding epicatechin values of 80.83 ± 12.99 mg g^−1^ for freeze-dried flour from Sangiovese grapes. These values are close to those found in our research, which used an oven with air circulation at 40, 50, and 60°C. Our results suggest that gallic acid, catechin, and epicatechin contents were not influenced by the choice of drying method. Moreover, there were no significant differences between most of the values in the formulations when related to catechin, except for EV3 and EV4.

There was a little variation in the catechin and epicatechin contents of formulation EV5. For free phenolic acids, the highest concentration of gallic acid was found in compound EV6. Based on the results, it was determined that the extracts included phenolic compounds in their composition. However, for resveratrol, myricetin, and quercetin, the obtained concentrations of the samples were relatively low. However, the presence of these compounds in most analyzed samples was identified.

**Figure 2:**
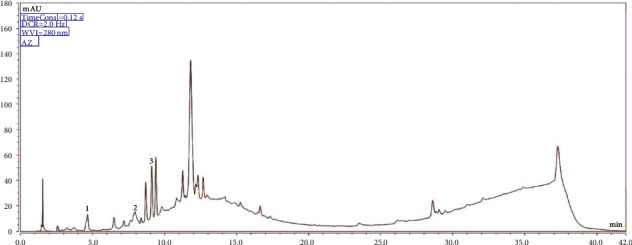
Chromatogram of formulation EV5 (1 = gallic Acid; 2 = catechin; 3 = epicatechin).

Thus, it was determined that the six formulations composed of grape peel and arrowroot contained these phytochemical compounds, but future studies can improve the sensitivity of this method. Notably, although these three compounds were not quantified, the chromatogram peaks suggested the presence of these compounds in the phytochemical composition of the extracts (Figure 2).

Flavanols are present in grapes, mainly in the forms of catechin, epicatechin, and proanthocyanidins, and accumulate in grape seeds and skin [55]. It was determined that the biological activities of flavonoids strongly depend on several factors, such as the degree of glycosylation and the type of sugar residue. The consumption of dietary flavonoids derived from grapes in the form of grape extracts and grape grain powder has been shown to effectively suppress oxidative stress and prevent oxidative damage in vivo [72, 88, 89]. These activities were attributed to the various functions of grape flavonoids as free-radical scavengers and metal chelating compounds [72, 88, 89].

Quercetin is the main flavonoid present in the human diet, with an estimated daily consumption between 50 and 500 mg [90]. From this complex group, the main constituents in grapes are catechin and epicatechin monomers. Compounds such as resveratrol, quercetin, catechins, and proanthocyanidins demonstrate several biological activities, including cardioprotective, anticancer, anti-inflammatory, and antimicrobial properties, mainly due to their antioxidant activity [91].

## 4. Conclusions

The results found in this study indicate that the Shiraz grape peel obtained from the waste of the wine industry in Vale do São Francisco, Bahia, Brazil, has the potential to be used as a source of fiber and phenolic and antioxidant compounds. The characterization of the six antioxidant compounds in powder developed from Shiraz grape skin and arrowroot showed that the drying method and dehydration temperature influenced the centesimal composition but had very little influence on their phenolic composition or antioxidant activity. Catechins, epicatechins, and quercetin were observed to be the phenolic compounds with the highest expressions. Evaluation of the antiradical activity of the oven-dried compounds indicated the excellent ability of DPPH to inhibit free radicals. Thus, the composition and low cost of these materials, along with the related dehydration process, may allow their use in the food industry, contributing to human nutrition, besides the environmental aspects related to the reduction of indiscriminate disposal of organic material.

## Figures and Tables

**Table 1:** Conditions of the drying processes used to obtain powdered antioxidant compounds.

Formulation	Temperature (°C)	Grape content (%, m/m)	Arrowroot content (%, m/m)
EV1	40	70	30
EV2	40	60	40
EV3	50	70	30
EV4	50	60	40
EV5	60	70	30
EV6	60	60	40

**Table 2:** Centesimal composition of Shiraz grape peels.

Centesimal composition	Means ± SD
Moisture (%)	68.82 ± 1.07
*A*_w_	0.870 ± 0.01
Ash (%)	4.12 ± 0.05
pH	3.54 ± 0.05
Total titratable acidity (%)	20.65 ± 0.08
Acidity in citric acid (%)	1.35 ± 0.01
Lipids (%)	1.48 ± 0.29
Proteins (%)	2.32 ± 0.33
Total soluble solids (°Brix)	1.90 ± 0.01
Hemicellulose	4.5 ± 0.66
Cellulose	13.4 ± 0.05
Lignin	15.3 ± 0.88

Results are expressed as the mean ± standard deviation.

**Table 3:** Colorimetric characterization of Shiraz grape peels.

Colorimetric	Means ± SD
*L* ^*∗*^	30.18 ± 0.70
*a* ^*∗*^	3.20 ± 0.43
*b* ^*∗*^	0.27 ± 0.07
*C* ^*∗*^	3.21 ± 0.43
*h*	4.74 ± 0.77

Results are expressed as the mean ± standard deviation.

**Table 4:** Bioactivity of the compounds in different samples.

Bioactive properties	Aqueous extract	Ethanloic extract 50%	Ethanloic extract 80%	Peels
Total phenolics (mg GAE g^−1^)	55.84 ± 3.74^c^	149.11 ± 9.27^b^	157.09 ± 6.96^a^	—
Flavonoids (mg QE g^−1^)	0.75 ± 0.50^c^	2.00 ± 0.09^a^	1.32 ± 0.89^b^	—
DPPH-IC_50_ (*μ*g mL^−1^)	0.73 ± 0.41^a^	0.17 ± 0.01^c^	0.21 ± 0.02^b^	—
Anthocyanins (mg g^−1^)	—	—	—	1.53 ± 0.41

Results are expressed as the mean ± standard deviation. Statistical analysis: values showing the same letter in the same line do not indicate significant differences (*p* > 0.05) based on a Tukey test at a 95% confidence level.

**Table 5:** Color analysis of powdered composites obtained by different process conditions in conventional kilns with air circulation.

Chroma	EV1	EV2	EV3	EV4	EV5	EV6
*L* ^*∗*^	46.87 ± 1.25^b^	51.43 ± 0.45^a^	47.64 ± 1.30^b^	47.95 ± 1.34^b^	45.82 ± 0.29^b^	46.46 ± 2.27^b^
*a* ^*∗*^	8.13 ± 0.32^a^	7.5 ± 0.05b	8.10 ± 0.08^a^	7.46 ± 0.07^b^	7.95 ± 0.16^ab^	7.54 ± 0.18^b^
*b* ^*∗*^	2.21 ± 0.03^ab^	2.02 ± 0.05^b^	2.36 ± 0.01^a^	2.08 ± 0.02^b^	2.44 ± 0.10^ab^	2.27 ± 0.08^ab^
*C*	8.42 ± 0.32^a^	7.76 ± 0.07^b^	8.44 ± 0.08^a^	7.75 ± 0.06^b^	8.32 ± 0.19^ab^	7.87 ± 0.19^b^
H°	15.23 ± 0.36^c^	15.07 ± 0.24^c^	16.24 ± 0.18^b^	15.56 ± 0.15^c^	17.09 ± 0.36^a^	16.75 ± 0.42^ab^
	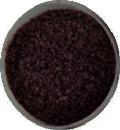	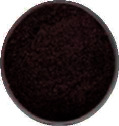	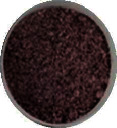	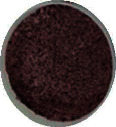	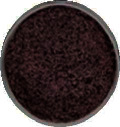	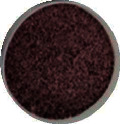

Results are expressed as the mean ± standard deviation. Statistical analysis: values showing the same letter in the same line do not indicate significant differences (*p* > 0.05) based on a Tukey test at a 95% confidence level.

**Table 6:** Total phenolics, total flavanols, DPPH radical scavenging activity, and total anthocyanins in powdered compost extracts from Shiraz (*Vitis vinifera* L.) and arrowroot (*Maranta arundinacea* L.).

Bioactive properties	EV1	EV2	EV3	EV4	EV5	EV6
Total phenolics (mg GAE g^−1^)	139.70 ± 0.02^a^	130.08 ± 0.02^b^	131.94 ± 0.02^b^	128.32 ± 0.02^c^	130.34 ± 0.02^b^	129.79 ± 0.02^bc^
Flavonoids (mg QE g^−1^)	4.01 ± 0.02^ab^	3.5 ± 0.02^c^	3.90 ± 0.02^b^	3.31 ± 0.02^c^	4.17 ± 0.02^a^	3.91 ± 0.02^b^
DPPH-IC_50_ (*μ*g mL^−1^)	0.17 ± 0.02^ab^	0.17 ± 0.02^ab^	0.19 ± 0.01^a^	0.16 ± 0.02^b^	0.18 ± 0.01^ab^	0.19 ± 0.02^a^
Anthocyanins (mg g^−1^)	1.16 ± 0.15^b^	1.47 ± 0.14^a^	1.49 ± 0.03^a^	1.46 ± 0.09^a^	1.19 ± 0.04^b^	1.43 ± 0.08^a^

**Table 7:** Identification and quantification of the phenolic compounds in different powdered antioxidant formulations by UHPLC.

Formulations	Gallic acid (mg g^−1^)	Catechin (mg g^−1^)	Epicatechin (mg g^−1^)
EV1	1.188 ± 0.061^a^	6.298 ± 1.124^a^	83.391 ± 18.870^a^
EV2	1.149 ± 0.023^a^	5.470 ± 1.609^a^	88.539 ± 2.994^a^
EV3	1.116 ± 0.110^a^	<LOQ	87.046 ± 1.262^a^
EV4	1.105 ± 0.024^a^	<LOQ	85.705 ± 3.538^a^
EV5	1.129 ± 0.039^a^	7.124 ± 0.130^a^	95.087 ± 1.138^a^
EV6	1.203 ± 0.054^a^	7.058 ± 0.399^a^	91.558 ± 0.230^a^

<LOQ: below the limit of quantification.

## Data Availability

The data supporting this study were described in the references list, with the respective DOI.
